# Honey bee colony losses and causes during the active beekeeping season 2022/2023 in nine Sub-Saharan African countries

**DOI:** 10.1371/journal.pone.0322489

**Published:** 2025-05-19

**Authors:** Beatrice T. Nganso, Workneh Ayalew, Abebe J. Wubie, Freweini Assefa, Lulseged Belayhun, Nelly N. Ndungu, Daniel Toroitich, Z. Ngalo Otieno-Ayayo, Mbatha B. Wambua, Yudah O. Oyieyo, Ntirenganya Elie, Rachidatou Sikirou, Souradji B. Idrissou, Willy Mwiza, S. Turner, Bridget O. Bobadoye, Sidonie T. Fameni, Sayemie Gaboe, Mawufe K. Agbodzavu, Patrick Mafwila, Geraud C. Tasse Taboue, Kimathi Emily, Tonnang Z.E. Henri, Saliou Niassy, Simplice N. Fonkou, Christian W. W. Pirk, Alison Gray, Robert Brodschneider, Victoria Soroker, Sevgan Subramanian

**Affiliations:** 1 Environmental Health Theme, International Centre of Insect Physiology and Ecology (icipe), Nairobi, Kenya; 2 Apiculture and Beneficial Insects Research Institute, Kenya Agricultural and Livestock Research Organization, Marigat, Kenya; 3 Department of Biological, Physical and Health Sciences, Rongo University, Rongo, Migori, Kenya; 4 Rwandan Association of Ecologists (ARECO Rwanda-NZIZA), Kigali, Rwanda; 5 Laboratory of Plant Protection (LADC), National Institute of Agricultural Research of Benin (INRAB), Cotonou, Benin; 6 Agriculture and Animal Resources Development Board (RAB), Kigali, Rwanda; 7 Malaika Honey Company, Kampala, Uganda; 8 Department of Forest Conservation and Protection, Forestry Research Institute of Nigeria (FRIN), Ibadan, Nigeria; 9 Department of Biological Sciences, Faculty of Science, University of Maroua, Maroua, Cameroon; 10 Nimba Beekeepers Incorporated, Nimba, Liberia; 11 International Institute of Tropical Agriculture (IITA), Kinshasa, Democratic Republic of Congo,; 12 Department of Animal Production, University of Kinshasa, Kinshasa, Democratic Republic of Congo,; 13 Multipurpose Research Station, Institute of Agricultural Research for Development, Bangangté, Cameroon; 14 University of KwaZulu-Natal, School of Agricultural, Earth, and Environmental Sciences, Pietermaritzburg, South Africa; 15 African Union Inter-African Phytosanitary Council (AU-IAPSC), Yaoundé, Cameroon; 16 Association for the Development of Agriculture, Fisheries and Animal Resources (ADAFAR), Yaoundé, Cameroon; 17 Social Insects Research Group, Department of Zoology and Entomology, University of Pretoria, Pretoria, South Africa; 18 Department of Mathematics and Statistics, University of Strathclyde, Glasgow, Scotland, UK; 19 Department of Biology, University of Graz, Graz, Austria; 20 Department of Entomology, Institute of Plant Protection, Agricultural Research Organization, the Volcani Center, Rishon LeZion, Israel; Ladoke Akintola University of Technology, NIGERIA

## Abstract

This study reports for the first-time a multi-country survey of managed honey bee colony loss rates and associated risk factors during the active beekeeping season 2022/2023 in nine Sub-Saharan African countries, namely Kenya, Ethiopia, Rwanda, Uganda, Benin, Liberia, Nigeria, Cameroon and Democratic Republic of the Congo. It also evaluates the sustainability of bee swarm catches as a primary source for expanding apiary size by African beekeepers. In this survey, the 1,786 interviewed beekeepers across these countries collectively managing 41,761 colonies registered an overall loss rate of 21.3%, which varied significantly among countries (from 9.7 to 45.3%) and hive types (from 10.6% in hives with movable frames to 17.9% in frameless hives). The perceived causes of losses in order of significance were issues beyond the beekeeper’s control (mostly theft, drought, and bushfire), absconding and pests (mostly wax moth, small and large hive beetles, ants and *Varroa destructor* mite), but this pattern varied greatly across countries. Among the management practices and characteristics, migratory beekeepers and professional beekeepers experienced lower losses than beekeepers practicing stationary beekeeping and semi-professionals and hobby beekeepers, respectively. Insights into the number of bee swarms caught revealed a significant decrease in swarm availability over the past three years in Kenya, while some regions in Ethiopia showed the opposite trend, requiring further investigation. Overall, this comprehensive survey highlights the complexities and challenges faced by beekeepers in Sub-Saharan Africa, underscoring the need for targeted interventions and sustained research to support the resilience and growth of the apicultural sector.

## Introduction

Over the past 17 years, countries in the Northern Hemisphere, particularly the United States [1–3], Europe [4–6], Canada [7–9] and Mexico [6,10–12], have consistently reported significant winter, summer and/or annual colony losses of the honey bee, *Apis mellifera* L. These losses have substantial economic implications for the apicultural and agricultural sectors as well as for the environment [13–15]. Several factors, which sometimes act synergistically, contribute to these honey bee colony losses [1,8,16–18]. These include invasive pests (especially *Varroa destructor*), pathogens (notably viruses associated with the *Varroa* mite), issues beyond the beekeepers’ control (such as pesticides, extreme weather conditions and natural disasters like flooding, fire or theft) and management practices (e.g., hive migration and queen replacement) [1,8,16–18]. The impact of these stressors on colony mortality varies by country and season [3,6].

In contrast to the situation in the Northern Hemisphere, reports suggest that colony losses in the Southern Hemisphere (e.g., Africa, South America and Australia) have not been severe [4,19–21]. However, this may not fully reflect the situation due to limited availability of long-term spatiotemporal surveys documenting managed honey bee colony losses and their causes in this part of the world [21,22]. For example, a few large-scale and spatiotemporal studies have reported annual colony losses exceeding 30% in several Latin American countries [23,24], while losses below 25% were reported in Australia [25] and New Zealand [6]. In the African continent, data on colony losses and causes are available only for a few countries in the south, north and east. In South Africa, for example, high total colony loss rates of 29.6% and 46.2% were reported during the active beekeeping seasons from September 1^st^ to April 1^st^ in 2009/2010 and 2010/2011, respectively [26]. This period corresponds to spring and summer in the Southern Hemisphere [27]. These losses were more severe in migratory than stationary beekeeping operations, and small hive beetles, *Varroa* mites, absconding and chalkbrood disease were identified as the key factors responsible for these losses [26]. In North Africa, Egypt experienced loss rates of 35.5% and 38.8% from September to March in 2011/2012 and 2012/2013, respectively, primarily due to *Vespa orientalis*, starvation, *Varroa* mites and poor quality of queens or loss of queens, referred to as queen problems in this review [28]. Meanwhile, recent reports showed lower colony loss levels in Egypt (24.3% in 2019/2020) and Algeria (9.8–12.2% in 2017/2018, 2018/2019 and 2019/2020) during the non-active season of beekeeping, with unresolvable queen problems (e.g., drone-laying queens) and natural disasters cited as the main factors [5–7]. High levels of losses (24.1–66.4%) were recently reported in two regions of Ethiopia during the 2022/2023 non-active season of beekeeping, primarily due to natural disaster (mostly war) and dead colonies or empty hives (which could be a result of absconding) [29]. These spatially limited studies contrast sharply with the broad scale of beekeeping across Africa, highlighting the huge gap in colony loss reporting on the continent.

Apiculture practices in Africa differ significantly from those in most parts of the world [30]. Of the many millions of colonies spanning eleven endemic honey bee subspecies on the continent [31,32], only a small fraction are managed bees [33]. Most African beekeepers predominantly rely on capturing bee swarms to sustain and expand their apiaries during the active beekeeping season, which is characterized by swarming, migration of colonies, absconding and honey harvesting, with minimal routine management [34–38]. Thus, the factors driving managed honey bee colony losses in Africa are likely to be distinct from those reported in the Western world, influenced not just by genetic and environmental factors but also by distinct management practices, cultural, and socio-economic factors. In fact, many African beekeepers utilize various hive types, movable or frameless structures [37,39], and typically do not select for traits such as low absconding/swarming or defensiveness, even though these behaviors are more pronounced in African subspecies compared to their European counterparts [31,40]. The adoption of routine apiary management practices such as hive inspection, requeening, pest control, and provision of water and/or supplementary feeding is minimal among African beekeepers [26,37]. Additionally, a lack of education and experience in good colony management and harvesting techniques tailored to specific hive types can exacerbate the absconding rate and/or colony mortality [35,37]. Furthermore, minor apicultural pests such as wax moths, large hive beetles and ants reportedly cause up to 50% loss of managed honey bee colonies in some parts of Africa [37,41]. Meanwhile, the impacts of the ectoparasitic *Varroa* mite and its associated viruses remain poorly documented in most African countries, and their significance in causing colony losses in the apicultural industry remain poorly studied, although in South Africa [19,42,43], Kenya [44–46] and Ethiopia [47], these impacts on managed honey bee colonies are considered insignificant.

In this multi-country study, we attempted for the first time to quantify and compare loss rates of managed honey bee colonies during the active beekeeping season in nine Sub-Saharan African countries. Additionally, we explored potential risk factors contributing to these losses. We further compared the colony loss rates based on several criteria: the country of operation, the type of beekeeping operation (professional, semi-professional or hobbyist), training in best beekeeping practices, migratory versus non-migratory beekeeping, the types of hives used by the beekeepers, and other colony management practices (provision of supplementary feeds and/or water at the onset of swarming to enhance colony establishment).

## Methods

### The survey

In this study, we adapted survey questionnaires developed by COLOSS (Prevention of honey bee COlony LOSSes; www.coloss.org) [5,6,10,48] to establish a standardized questionnaire that better suits the specific conditions of beekeeping in Africa. The original COLOSS questionnaire is designed for the non-active beekeeping season (referred to as winter, which spans from October 1^st^ to March 31^st^) [5,6,10,48]. However, our study focused on the active beekeeping season in the surveyed African countries, which spanned from September 1^st^ 2022 to June 30^th^ 2023. During this period, the total number of managed colonies fluctuated, as many African beekeepers place empty hives on trees and other standing structures to capture migrating honey bee swarms and expand their apiaries.

The English version of the adapted questionnaire was distributed to national coordinators from Eastern (Kenya, Ethiopia, Rwanda, and Uganda), West (Benin, Liberia, and Nigeria) and Central (Cameroon and the Democratic Republic of the Congo) Africa, who led the survey. These national coordinators, selected from local research organizations and/or private sectors, were responsible for collecting responses from beekeepers. They conducted in-person interviews and obtained verbal consent from the respondents before data recording. Additionally, the coordinators from Kenya, Rwanda and Benin distributed the questionnaires to the beekeepers during workshops and meetings and followed up by phone calls to complete the data collection. When necessary, the questionnaire was translated into French and/or local languages during the data collection process. Prior to data collection, this study was reviewed by the science committee at the International Centre of Insect Physiology and Ecology (*icipe*) in Nairobi, Kenya and a written ethics approval letter was obtained from the committee.

As in the COLOSS questionnaire [5,6,10,48], the questions (Q) in our survey were categorized as mandatory (marked with an asterisk) or optional (without an asterisk) (see Table S1). The mandatory questions focused on disease symptoms and the loss rate of production colonies, defined as colonies with a queen that can provide a honey harvest during the active beekeeping season. We also evaluated the impact of beekeeping experience, training in best beekeeping practices, beekeeping activity, colony management and hive types on colony loss rates.

The national coordinators calculated the colony loss rate in **Q11** during the interview using the following formula: [(**Q7** + **Q8**) – (**Q9** + **Q10**)]. For losses attributed to pathogens, the national coordinators carefully explained the symptoms of viral diseases, microsporidia, and bacterial diseases to the beekeepers, as most of them were unfamiliar with and not knowledgeable about these topics. Question 18 (**Q18)** was only added to the questionnaire in June 2023, but some national coordinators were able to revisit the beekeepers to gather this information.

### Statistical analyses

All statistical analyses were conducted using R software version 4.3.2 [49]. Before estimating the total loss rate per country, the validity of the data for each individual beekeeper was first checked. The following criteria were applied to ensure data accuracy: (a) the number of colonies at the start of swarming should be indicated and must be greater than zero; (b) the number of colonies lost should be indicated and must be greater than or equal to zero; and (c) the total loss rate attributed to various risk factors (issues beyond the beekeeper’s control, absconding, starvation, pesticide poisoning, queen problems, pests/pathogens, and unknown symptoms) should not exceed the number of colonies lost reported by a beekeeper. Only data that met these criteria were included in the calculation of the overall loss rate for each of the nine surveyed countries. To compute the 95% confidence interval (CI) for the overall loss rate, the quasi-binomial family of generalized linear models (GLMs) was fitted using the R codes available in the Standard Survey Methods chapter of the COLOSS BEEBOOK [50] and the significance level alpha was set at 0.05. This same statistical approach was used to determine how factors such as the country, beekeeping type (professional, semi-professional, or hobbyist), hive types (movable frames or frameless) and beekeeping activity (migratory or non-migratory) influenced the overall loss rate. We also estimated the effect of the individual factors (e.g., the beekeeping type, hive types, and beekeeping activity) mentioned above within countries such as Ethiopia, Kenya, and Benin, where more than 90 valid survey data responses were available. Additionally, the relative risk of loss for each region within a country was calculated as the overall loss rate for that region divided by the overall loss rate of all regions, as described in Gray et al. [6]. The results were visualized on maps obtained from Natural Earth (https://www.naturalearthdata.com/) with administrative shapefiles sourced from GADM (https://gadm.org/). A Poisson GLM fitted with a log link function was also used to analyze changes in the number of bee swarms caught over the past two years in Ethiopia and Kenya. This statistical approach was also used to compare swarm catches over time between Ethiopia and Kenya as well as among regions within Ethiopia. Means and t-intervals are quoted for the numbers of colonies managed in the different countries and for various subgroups of beekeepers. Benin was not included in this analysis because beekeepers did not provide sufficient data on swarm numbers due to difficulties in recalling this information for the previous periods (2021/2022 and/or 2020/2021), prior to the current survey conducted in 2022/2023.

## Results

### Loss rates of managed honey bee colonies across the nine surveyed countries

**Fig 1:**
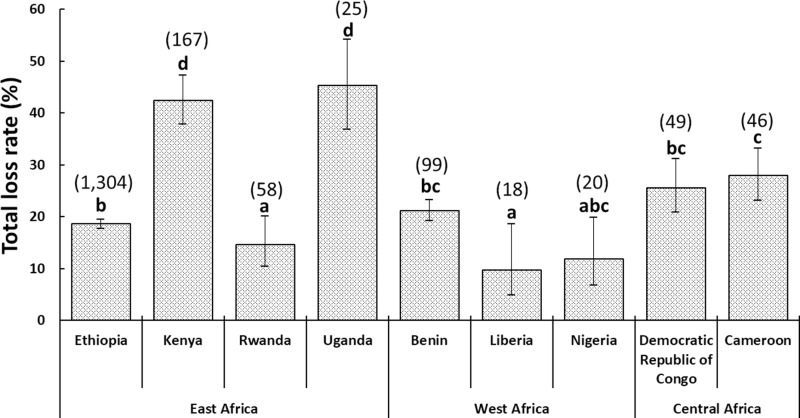
A bar graph showing the overall colony loss rates and 95% confidence intervals for the 1,786 beekeepers interviewed across the nine Sub-Saharan African countries. Different letters above the bars indicate significant differences among the countries when compared using the quasibinomial GLM with “country” as an explanatory factor as in van der Zee et al. [50], *p* < 0.05. The numbers in brackets above the bars represent the total number of interviewed beekeepers who provided valid data for each country.

A total of 1,786 beekeepers from nine surveyed African countries provided valid loss data for 41,761 honey bee colonies managed during the active beekeeping season (Fig 1). This number of respondents represents less than one percent of the total number of beekeepers in the nine surveyed countries. Among the respondents, 95.3% relied on swarm catches for expanding their operations, whereas only 4.7% practiced queen rearing for the same purpose.

Following the honey harvest, the 1,786 beekeepers ended up with 32,861 colonies out of the initial 41,761, resulting in a total loss of 8,900 colonies. This represents a total loss rate of 21.3% (95% CI: 20.4–22.2%). The loss rate varied significantly among the surveyed countries (quasibinomial GLM: F = 41.5, df = 8, *p* < 0.001) (Fig 1). In fact, Uganda and Kenya registered the highest loss rates, whereas Liberia, Nigeria and then Rwanda registered the lowest. Loss rates in Ethiopia and Cameroon were intermediate.

### Loss rates of managed honey bee colonies within Ethiopia, Kenya and Benin, and regional risk of losses across all participating countries during the active beekeeping season

**Fig 2:**
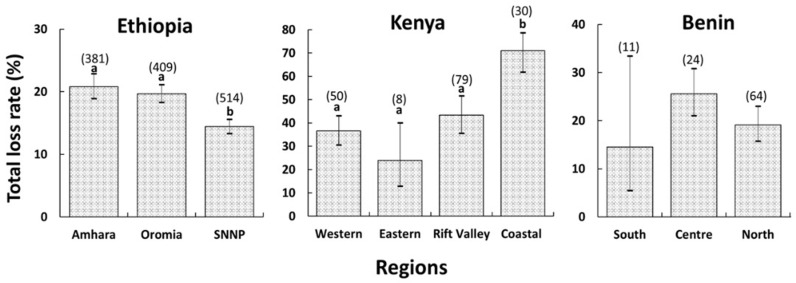
Bar graphs showing the total loss rates and 95% confidence intervals for each region in Ethiopia, Kenya, and Benin. Different letters above the bars indicate significant differences among the regions when compared using the quasibinomial GLM with region as an explanatory factor as in van der Zee et al. [50], *p* < 0.05. The numbers in brackets above the bars represent the total number of interviewed beekeepers who provided valid data for each region.

**Fig 3:**
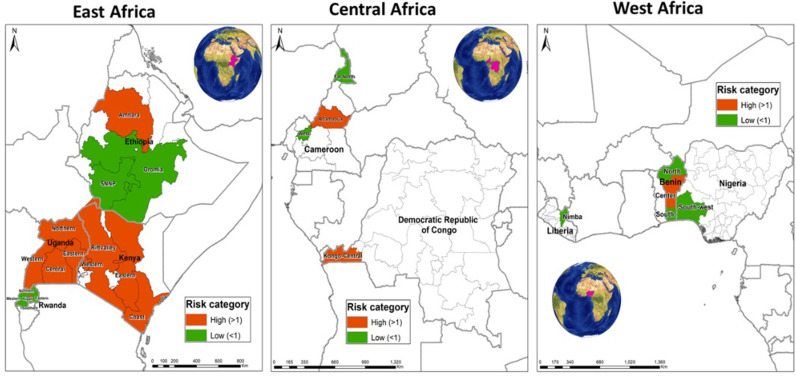
Map with color coding showing the relative risk of loss at the regional level for each country in Sub-Saharan Africa. Red and green colors indicate regions with a relative risk of loss higher and lower than one, respectively. All regions considered for this analysis had valid answers from at least three beekeepers. Regions where the survey was not carried out have not been marked.

The total loss rate also showed significant variations among regions within Ethiopia (quasibinomial GLM: F = 19.7, df = 2, *p* < 0.001) (Fig 2A) and Kenya (quasibinomial GLM: F = 7.3, df = 3, *p* < 0.001) (Fig 2B), but not in Benin (quasibinomial GLM: F = 3.02, df = 2, *p* = 0.05) (Fig 2C). In Ethiopia, beekeepers in the Southern Nations, Nationalities, and Peoples' (SNNP) region registered lower losses than those from the Amhara and Oromia regions, while in Kenya, those from the Coastal region registered higher losses than those from the other three regions. Additionally, regions of higher and lower risk of losses were observed within each of the nine participating countries when calculating the relative risk of the total colony losses at country level (Fig 3).

### Patterns of risk factors associated with colony losses across the nine surveyed countries

**Fig 4:**
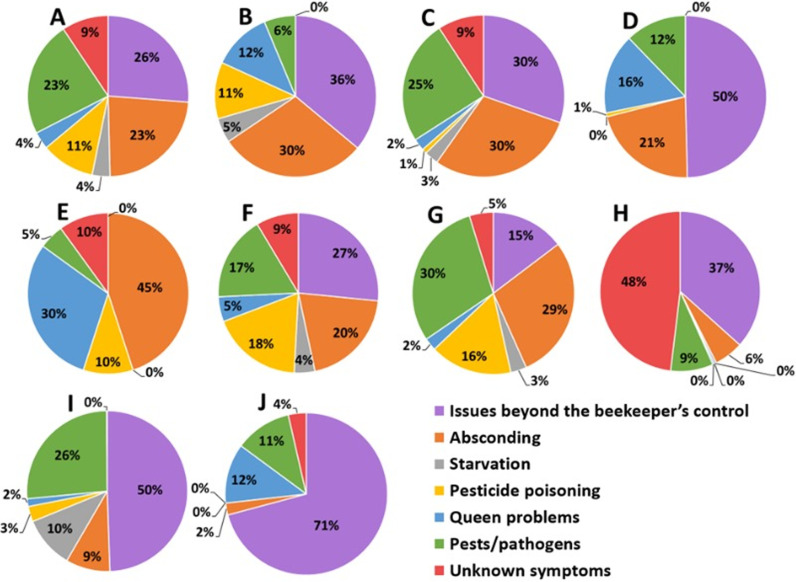
Pie charts showing the different patterns of risk factors associated with total colony losses. (**A**) across all surveyed countries and in the individual countries, including Uganda **(B)**, Kenya **(C)**, Liberia **(D)**, Nigeria **(E)**, Rwanda **(F)**, Ethiopia **(G)**, Cameroon **(H)**, Benin (**I**) and the Democratic Republic of Congo **(J)**.

The general analysis of the beekeeper-perceived causes of colony losses across the nine surveyed countries revealed issues beyond the beekeeper’s control (theft, drought, and bush fire) as the most significant, followed, in descending order of significance, by absconding, pests/pathogens (wax moth, small and large hive beetle, ants, *Varroa* mite and *Nosema*), and pesticide poisoning (Fig 4A). However, this order of risk factors varied by country. For example, in Uganda, the main factors were issues beyond the beekeeper’s control (mostly theft) first, followed by absconding, queen problems (mostly queen loss) and pesticide poisoning (Fig 4B). On the other hand, the three leading causes of colony loss in Kenya were issues beyond the beekeeper’s control (mostly theft and drought), absconding, and pests (wax moth, small and large hive beetles, ants and *Varroa* mite) (Fig 4C).

In Liberia, issues beyond the beekeeper’s control (mostly theft), absconding, queen problems (mostly queen loss) and pests (ants) were the primary causes of losses (Fig 4D). The six Liberian beekeepers who reported ant attacks treated their colonies against this pest by applying waste engine oil on hive stands. In Nigeria, absconding and queen problems (mostly queen loss) mainly accounted for the losses (Fig 4E), whilst in Rwanda issues beyond the beekeeper’s control (mostly theft), absconding, pesticide poisoning, and pests (mostly ants) were mainly responsible for the losses (Fig 4F).

In Ethiopia, the leading causes of colony losses were pests/pathogens (wax moth, small and large hive beetle, ants, *Varroa* mite and *Nosema*), absconding, pesticide poisoning, and issues beyond the beekeeper’s control (mostly theft) (Fig 4G). In Cameroon, issues beyond the beekeeper’s control (mostly theft) dominated, but most beekeepers could not specify the factors responsible for the losses (Fig 4H). Issues beyond the beekeeper’s control (mostly theft) and pests (mostly ants) dominated in Benin (Fig 4I). In the Democratic Republic of Congo, issues beyond the beekeeper’s control (mostly theft and bush fire), queen problems (mostly queen loss) and pests (mostly ants and termites) were the leading causes of losses (Fig 4J). Beekeepers in this country often used fresh wood ash, grease, or oil (e.g., vegetable oil or waste engine oil) on hive stands against ants and termites.

### Patterns of risk factors associated with colony losses within Ethiopia, Kenya, and Benin

**Fig 5:**
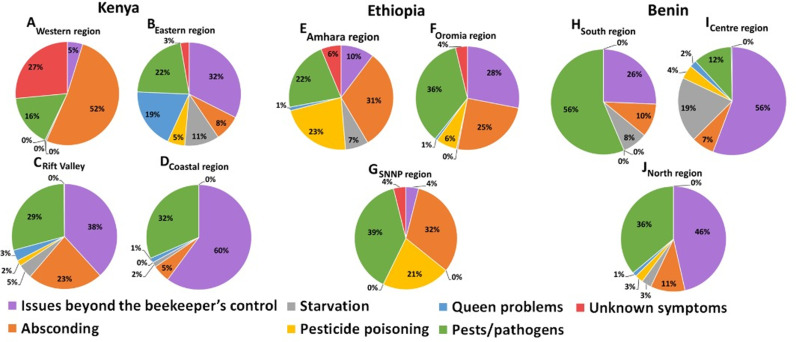
Pie charts showing the different patterns of risk factors associated with total colony losses by regions within Kenya **(A-D), Ethiopia (E-G) and Benin** **(H-J)**.

In this study, the pattern of risk factors also varied by region within the country. For example, in the Western region of Kenya, absconding (52%) and pests (16%) (mostly wax moth and the small hive beetle) were major contributors to colony losses (Fig 5A), whereas issues beyond the beekeeper’s control (32%; mostly theft), pests (22%; mostly wax moth) and queen problems (19%; mostly queen loss) dominated in the Eastern region of the country (Fig 5B). On the other hand, issues beyond the beekeeper’s control (38%; mostly theft), pests (29%; mostly wax moth) and absconding (23%) dominated in the Rift Valley region (Fig 5C), whereas in the Coastal region, issues beyond the beekeeper’s control (60%; mostly drought) and pests (32%; mostly the large hive beetle and the wax moth) largely explained colony losses (Fig 5D). Only 24 of the 87 Kenyan beekeepers who reported pest infestations managed some of these biotic stressors inside their colonies. Although these Kenyan beekeepers did not treat their colonies specifically against *Varroa* mite, wax moths and small and large hive beetles, they used an insecticide called “Sevin Dudu Dust” with an active ingredient of 7.5% Carbaryl against ants and applied grease or waste engine oil to hive stands for ant prevention.

In the Amhara region of Ethiopia, absconding was the major cause accounting for 31% of losses, followed by pesticide poisoning at 23%, and pests/pathogens at 22% (Fig 5E). The major pests and pathogens reported were wax moth and the small hive beetles followed, in descending order, by *Varroa* mite, *Nosema* and the large hive beetle. The presence of excreta (diarrhea) on hive components was indicative of *Nosema* infection [51,52]. It is worth noting that the respondents reported 31 colonies lost due to nosemosis out of the 8,973 total managed colonies from this region, representing a total loss rate of just 0.3% out of the 20.8% overall loss rate reported for the Amhara region. In the Oromia region of Ethiopia, on the other hand, pests (36%), issues beyond the beekeeper’s control (28%; mostly theft) and absconding (25%) dominated (Fig 5F). The major reported pests in this region included wax moths, followed by small hive beetles and *Varroa* mites. In the SNNP region, pests (39%), absconding (32%) and pesticide poisoning (21%) dominated (Fig 5G). Beekeepers from this region reported wax moths, followed by *Varroa* and the small hive beetle, as their major pests. Whilst Ethiopian beekeepers did not treat their colonies against *Varroa* mite and nosemosis, most of them practiced good apiary hygiene against wax moth, small and large hive beetle infestations, and used fresh wood ash on hive stands against ants.

When looking at the pattern of risk factors associated with the colony loss rate across the regions in Benin, our results revealed that pests (56%; mostly small hive beetle, ants and *Varroa* mite) and issues beyond the beekeeper’s control (26%; mostly theft) dominated in the Southern region of the country (Fig 5H). In the Central region, issues beyond the beekeeper’s control (56%; mostly theft) and starvation (19%) mainly explained the losses (Fig 5I), whereas issues beyond the beekeeper’s control (46%; mostly theft) and pests (36%; mostly ants) dominated in the Northern region (Fig 5J). In general, most beekeepers in Benin who reported problems with ants used permethrin insecticide and waste engine oil to treat against the ants.

### Impact of management and hive types on colony losses across the surveyed countries

Our results showed that beekeeping type significantly influenced the total loss rate across the nine participating countries (quasibinomial GLM: F = 37.2, df = 2, *p* < 0.001), with professional beekeepers losing fewer colonies (14.3% (95% CI: 11.0–18.3%)) than semi-professionals (22.8% (95% CI: 21.8–23.7%)) and hobbyists (39.6% (95% CI: 31.5–48.4%)). Most respondents (94%) were semi-professionals, while 5% and 1% were professionals and hobbyists, respectively. On average, professional beekeepers managed 97.0 ± 31.7 (95% CI) colonies, compared to 19.7 ± 1.1 (95% CI) and 19.9 ± 16.2 (95% CI) colonies managed by semi-professionals and hobbyists, respectively. Migratory beekeepers (39.5%) (17.9% (95% CI: 16.7–19.2%)) had a significantly lower colony loss rate than stationary ones (60.5%) (23.2% (95% CI: 22.0–24.5%)) across the nine participating countries (quasibinomial GLM: F = 30.7, df = 1, *p* < 0.001). These beekeepers moved their colonies during the active beekeeping season, mainly for crop pollination and honey production. The pollinated crops were mainly sunflower, coffee, sorghum, avocado, orange, mango, pawpaw, niger seed, and macadamia.

In this survey, the respondents who received training on best beekeeping practices (79% of the beekeepers) lost significantly fewer colonies (20% (95% CI: 19.0–21.0%)) than those who did not (21%) (26.1% (95% CI: 24.0–28.4%)) (quasibinomial GLM: F = 29.3, df = 1, *p* < 0.001). Among those, the majority of the interviewed beekeepers (74.5%) provided feeds to their colonies after honey harvest, while beekeepers (6%) who provided supplementary feeds to their colonies at the onset of the swarming season had a similar loss rate of 18.7% (95% CI: 17.9–19.6%)) to those who did not (94%) (26.1% (95% CI: 24.0–28.4%)) (quasibinomial GLM: F = 2.2, df = 1, *p* = 0.13). In both scenarios, the beekeepers who practiced supplementary feeding received training on best beekeeping practices and fed their colonies with different sugar sources (e.g., sugar syrup, Bee Fonda, mango, papaya, pineapple, and/or banana juices) and/or pollen substitutes (e.g., cassava, maize, sorghum, roasted pea, bean, soya, and/or barley flours). In water scarcity areas, beekeepers (82%) who provided water to their colonies registered significantly lower losses of 20.4% (95% CI: 19.5–21.4%) than those who did not (18%) (23.2% (95% CI: 20.9–25.7%)) (quasibinomial GLM: F = 8.1, df = 1, *p* < 0.01). Supplementary feeding and/or provisions of water were most practiced by beekeepers from Ethiopia and Rwanda, whereas those from Liberia and Nigeria practiced neither of these. Lastly, colony losses were significantly lower in movable frame hives (10.6% (95% CI: 9.7–11.5%)) compared to local frameless hives across the nine African countries (17.9% (95% CI: 16.5–19.3%)) (quasibinomial GLM: F = 120.8, df = 1, *p* < 0.001), with over 90% of colony migrations being carried out by beekeepers who had movable frame hives.

### Impact of management and hive types on colony losses within Ethiopia, Kenya, and Benin

In this survey, professional beekeepers lost fewer colonies than semi-professionals and hobbyists in Kenya (quasibinomial GLM: F = 10.4, df = 1, *p* < 0.01) and Benin (quasibinomial GLM: F = 4.9, df = 2, *p* < 0.01), but not in Ethiopia (quasibinomial GLM: F = 0.7, df = 1, *p* = 0.4). Most respondents in Ethiopia (98.8%), Kenya (93%) and Benin (84%) were also semi-professionals. In Ethiopia, semi-professional beekeepers, managed, on average, 17.4 ± 0.6 (95% CI) colonies, which was nearly half as low as the corresponding number for professionals (30.1 ± 5.8 (95% CI)). Similarly, professional beekeepers in Kenya, on average, managed 29.2 ± 20.3 (95% CI) colonies compared to 16.6 ± 3.6 (95% CI) colonies managed by semi-professionals. In Benin, professional beekeepers on average managed 153.4 ± 160.4 (95% CI) colonies compared to 22.9 ± 4.0 (95% CI) and 14.8 ± 10.3 (95% CI) colonies managed by semi-professionals and hobbyists, respectively.

In Ethiopia, the migratory beekeepers (45%) registered significantly lower colony losses (16.8% (95% CI: 15.8–17.9%)) than stationary beekeepers (55%) (19.7% (95% CI: 18.4–21%)) (quasibinomial GLM: F = 10.2, df = 1, *p* < 0.01). On average, these migratory beekeepers, who were all semi-professionals, managed fewer colonies (15.4 ± 0.6 (95% CI)) than stationary ones (19.3 ± 1.0 (95% CI)). In contrast, migratory and stationary beekeepers had similar losses in Kenya (quasibinomial GLM: F = 1.2, df = 1, *p* = 0.3) and Benin (quasibinomial GLM: F = 0.5, df = 1, *p* = 0.5). Our results also revealed that the 81%, 77% and 92% of respondents who received beekeeping training in Ethiopia (quasibinomial GLM: F = 3.4, df = 1, *p* = 0.1), Kenya (quasibinomial GLM: F = 0.4, df = 1, *p* = 0.5) and Benin (quasibinomial GLM: F = 0.01, df = 1, *p* = 0.1), respectively, had similar loss rates to those who did not receive any training.

Supplementary feeding at the onset of swarming had no significant impact on colony loss rate in Ethiopia (quasibinomial GLM: F = 0.2, df = 1, *p* = 0.6), Kenya (quasibinomial GLM: F = 0.2, df = 1, *p* = 0.6) and Benin (quasibinomial GLM: F = 0.2, df = 1, *p* = 0.6). All the participants from these countries fed their colonies after honey harvest. Additionally, all the Ethiopian beekeepers interviewed provided water to their managed colonies. Meanwhile, the loss rate of Kenyan beekeepers who provided water (32.9%) (quasibinomial GLM: F = 0.1, df = 1, *p* = 0.8) was not significantly different from that of beekeepers who did not (67.1%). Intriguingly, the 45% of beekeepers in Benin who provided water to their colonies had a significantly higher loss rate of 25.8% (95% CI: 21.9–30%) than the 55% of beekeepers who did not (16.3% (95% CI: 13–20.4%) (quasibinomial GLM: F = 11.7, df = 1, *p* < 0.001). Lastly, the loss rate in the local hives (16.1% (95% CI: 15.4–16.9%)) was approximately three times as high as that in the modern hives in Ethiopia (5.9% (95% CI: 5.5–6.5%)) (quasibinomial GLM: F = 481.4, df = 1, *p* < 0.001).

### Increase and decrease in the number of bee swarm catches over the past three years in Ethiopia and Kenya

**Fig 6:**
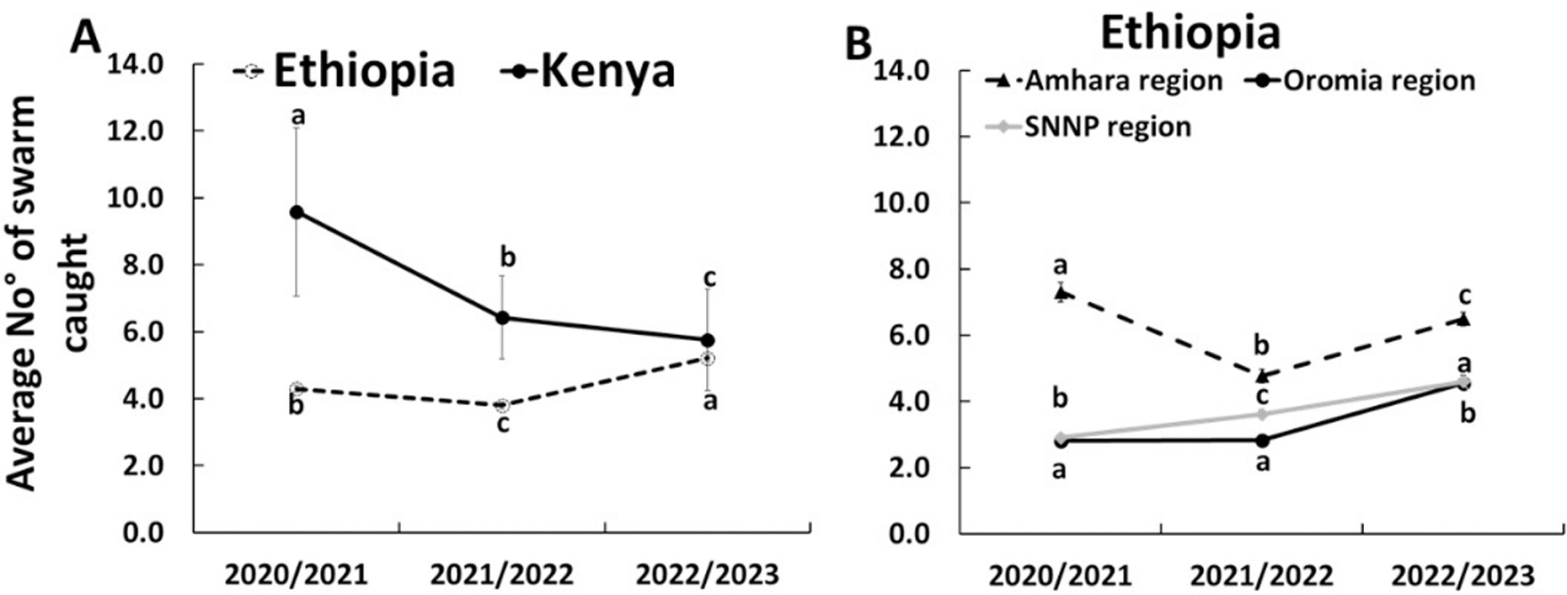
Line graph showing the increase or decrease in the average number of bee swarms caught over the past three years (Mean **± SE) in Ethiopia and Kenya (A) and in Amhara, Oromia and SNNP regions of Ethiopia** **(B)**. Different letters above the bars indicate significant differences among the years within the region when compared using the Poisson GLM with log link.

In Kenya, as shown in Fig 6A, the number of bee swarms caught over the past three years decreased significantly by about one third (from 9.6 ± 5.7 in 2020/2021 to 5.7 ± 1.5 in 2022/2023) (Poisson GLM: F = 18.2, df = 2, *p* < 0.001). The major decrease occurred between 2020/2021 (9.6 ± 5.7) to 2021/2022 (6.4 ± 1.2). In contrast, there has been a significant increase in the number of bee swarm catches over the past three years in Ethiopia (from 4.3 ± 0.1 in 2020/2021 to 5.2 ± 0.1 in 2022/2023) (Poisson GLM: F = 99, df = 2, *p* < 0.001). When comparing the number of bee swarm catches between Ethiopia and Kenya over the past three years, we found a significant effect of year (Poisson GLM: F = 88.7, df = 2, *p* < 0.001), country (Poisson GLM: F = 142.9, df = 1, *p* < 0.001) and the interaction between these two factors (Poisson GLM: F = 28.5, df = 2, *p* < 0.001). In fact, bee swarm catches were about one and half times as high in Kenya than in Ethiopia for 2020/2021 and 2021/2022, though were similar in 2022/2023 (Fig 6A). The number of bee swarm catches also differed between the surveyed regions of Ethiopia (Fig 6B). While a significant decrease (a drop of about one third) in the number of bee swarm catches was observed in the Amhara region of Ethiopia from 2020/2021 (7.3 ± 0.3) to 2021/2022 (4.8 ± 0.2) (Poisson GLM: F = 80.7, df = 2, *p* < 0.001), the opposite was observed in the Oromia region. In this region, the number of bee swarms caught increased significantly by approximately one and half times in 2022/2023 (4.6 ± 0.2) compared to both 2021/2022 and 2020/2021 (2.8 ± 0.1) (Poisson GLM: F = 58.7, df = 2, *p* < 0.001). Similarly, the number of bee swarms caught differed significantly between the years in the SNNP region (Poisson GLM: F = 76.7, df = 2, *p* < 0.001), increasing by approximately one- or 1.5-fold in 2022/2023 (4.6 ± 0.1) compared to 2021/2022 (3.6 ± 0.2) or 2020/2021 (2.9 ± 0.2). Similarly, when comparing the number of bee swarm catches among regions in Ethiopia over the past three years, we found a significant effect of year (Poisson GLM: F = 99.0, df = 2, *p* < 0.001), region (Poisson GLM: F = 421.3, df = 1, *p* < 0.001) and the interaction between the year and the region (Poisson GLM: F = 58.6, df = 2, *p* < 0.001). Bee swarm catches were not the same across regions and were higher in Amhara than Oromia and SNNP regions over the past two years.

## Discussion

### Trends in bee swarm availability in Kenya and Ethiopia and their implications for the sustainability of apiculture

The sustainability of apiculture and pollination services depends on beekeepers’ ability to maintain a stable number of productive colonies over time. This survey revealed that most interviewed beekeepers (95.3%) rely on capturing bee swarms to offset colony losses and expand their apiary size, as was reported previously for Africa [37]. Over the past three years, there has been a significant decrease in bee swarm availability in Kenya and to a lesser extent in the Amhara region of Ethiopia. An opposite trend over the same length of time was observed in the Oromia and SNNP regions of Ethiopia (Fig 6B). This disparity is likely to reflect regional differences in the environmental factors known to affect the number of wild and managed honey bee populations, which are the sources of bee swarms. These environmental stressors including land use change, pesticides and climate change have recently been identified as important contributors to pollinator decline in Africa [53].

The decline in swarm availability in Kenya was initially reported in 2010 by Muli et al. [54]. Similar observations were made between 2005 and 2006 in the Amhara region of Ethiopia, primarily due to farmland expansion and pesticide use [55]. Our findings further support the role of pesticide poisoning in colony losses in Ethiopia (Fig 4G), particularly in the Amhara and SNNP regions (Fig 5 E&G). Pesticide use is common in these regions due to extensive cereal, vegetable, and fruit cultivation [56]. The negative impact of pesticides such as organochlorines (e.g., endosulfan and dichlorodiphenyltrichloroethane (DDT)) and neonicotinoids (e.g., imidacloprid, thiamethoxam, acetamiprid) on beneficial insects, has been documented in Ethiopia [57–60], Kenya [61–63] and other parts of Africa [64,65], while most of these pesticides are already banned in other parts of the world [66,67]. Given these findings on significant change in swarm availability in Kenya and Ethiopia, long-term spatio-temporal studies are essential across African countries to better understand the dynamics of swarm availability and identify key drivers of change. Such research will be essential for informed decision-making and the development of strategies to support sustainable apiculture.

### Variation in honey bee colony loss rates across and within Sub-Saharan African countries

Regarding the losses, our study revealed a significant variation in honey bee colony loss rates among the nine participating African countries during the active beekeeping season, which corresponds to the spring and summer periods in the Southern Hemisphere [27]. Loss rates ranged from 9.7–45.3%. The highest loss rates, between 42% and 45%, were observed in Kenya and Uganda, while the lowest rates, from 9.7% to 14.6%, were found in Liberia, Nigeria, and Rwanda. The overall loss rates also differed considerably among regions within Ethiopia and Kenya and were in the range of 14.4–20.8% and 23.9–71%, respectively. The range of total colony losses recorded herein was similar to those reported for South African honey bees during the active beekeeping season (29.6–46.2%) [26]. It is important to note that respondents expressed concerns about their levels of losses, as these threatened the viability of their beekeeping businesses. The considerable variations in the above loss rates among African countries may be attributed to external causes (e.g., issues beyond the beekeeper’s control, such as pesticides), and internal factors (e.g., absconding, pests, colony management practices and sociological factors) [26,68–70], discussed below.

### External and internal risk factors associated with colony losses across and within Sub-Saharan Africa

Issues beyond the beekeeper’s control such as, theft, drought, and bushfires, emerged as primary causes, with varying impact by country and region. For example, theft was most pronounced in the Democratic Republic of Congo (71%) (Fig 4J), with no impact in Nigeria (Fig 4E). Potential solutions to mitigate thefts in the future include the development of owner-specific branded hives/frames, provision of hive insurance, development of state laws imposing heavy penalties for stealing beehives, and/or investment in anti-theft technologies such as GPS trackers and/or surveillance cameras, as was suggested for similar cases in South Africa [70]. A prolonged drought in 2022 severely affected apiculture in Kenya’s semi-arid regions (i.e., Eastern, Rift Valley and Coastal regions) and the Central and Northern regions of Benin, reducing forage and water availability for honey bees [71] and increasing bush fire risks [72]. Bush fires, often caused by slash-and-burn agriculture to prepare for the next planting season, was common in the Democratic Republic of Congo. Bush fire can have two impacts on apiculture: a direct impact by killing colonies and an indirect impact by eliminating their food sources. Farmers in developing countries in Asia and Africa still practice slash-and-burn agriculture [73]. While climatic events like drought are unpredictable [74,75], strategies to mitigate this issue include promoting sustainable agricultural practices that reduce the reliance on slash-and-burn methods, enhancing early warning systems for drought and fire, and improving water and supplemental food resource management to support apiculture during dry periods [76].

Absconding was a major cause of colony losses in this study, highest in Nigeria (45%; Fig 4E) and lowest in the Democratic Republic of Congo (2%; Fig 4J). Absconding, a natural migration of an entire colony to another site, was also identified as a cause of beekeepers’ colony losses during the active beekeeping season in South Africa [26]. In fact, these colonies are not lost to the ecosystem. Absconding is considered as a pronounced genetically based behavior of the African honey bee subspecies in response to stress caused by various disturbances including pests and predation, human manipulation and scarcity of resources [31,40,77], but the tendency to abscond varies between different subspecies [37]. For instance, some pests contributing to colony losses in this study were previously reported to elicit colony absconding. These include the wax moth [35,78], the small hive beetle [79], the large hive beetle [80] and ants [36,81]. Of note, attacks on hives by large hive beetles was widespread in the Coastal region of Kenya, where exceptionally high infestation levels of this pest, have recently been documented [37,80]. On the other hand, ants were predominantly observed in Rwanda, Cameroon, the Democratic Republic of Congo, the Amhara and Oromia regions of Ethiopia, and the Southern and Northern regions of Benin. Additionally, environmental factors such as drought conditions, as reported above, and starvation of bees as perceived by beekeepers in the Central region of Benin, may also trigger the relocation of honey bee colonies to areas with more favorable forage resources, as seen in some ant species [82]. Furthermore, even routine human activities such as colony examinations can precipitate absconding [77,83], highlighting the complex interplay of biotic and abiotic stresses impacting bee colony stability.

Regarding the role of pests and pathogens in colony losses, the respondents mainly reported pests visible to the naked eyes, with *Nosema* being the only pathogen reported, but this was detected indirectly by identification of fecal spots at the hive entrance [51,52], and was not confirmed through microscopic or molecular analysis. However, the reported occurrence of *Nosema* was confined to managed colonies in the Amhara region of Ethiopia. Previous studies have documented *Nosema* infection caused by *N. apis* in Ethiopia [84–86], but the extent to which it affects the health of honey bees still remains largely unclear in Ethiopia.

In general, most of the pests and predators reported as risk factors are considered benign to African honey bee colonies [19]. For instance, *Varroa* mite was so far reported as not significant in a few of the surveyed countries such as Kenya [45–47], Ethiopia [47] and Uganda [87]. This is also true in South Africa [88] and in Tunisia [89]. However, the impact of *Varroa* mite on managed honey bee colonies in other surveyed countries (e.g., Rwanda, Cameroon, Democratic Republic of Congo, Benin, Nigeria, and Liberia) remains unknown. Nevertheless, the beekeepers in most surveyed countries mentioned it among other common and conspicuous pests such as wax moths, ants, small and large hive beetles, as threats to their business. However, this could be a misattribution, blaming visible signs rather than underlying causes. In particular, small hive beetles and wax moths, which scavenge on abandoned hive resources, are often mistakenly identified as the primary cause of colony losses rather than a symptom of management issues. Such perceptions among the respondents may be related to poor apiary management practices, as over 90% of them are part-time (semi-professional) beekeepers who lose more colonies compared to their full-time (professional) counterparts. This observation suggests a potential association between beekeeping engagement level and pest impact, warranting further investigation to clarify these dynamics and improve colony management strategies. In fact, the adoption of good apiary hygiene practices generally helps to minimize damages and colony losses attributed to pests such as the wax moth, ants, small and large hive beetles [19,78,90]. Overall, these findings suggest that many of the survey participants have limited knowledge concerning the identification and management of pathogens that can compromise honey bee health and productivity. This knowledge gap underscores the urgent need to enhance local capacities in pathogen diagnosis and good beekeeping practices to safeguard the health of African honey bees. Such initiatives are crucial for the sustainable development of apiculture in Africa. Although the survey did not explicitly capture the impact of hive types on colony losses due to these pests, our results indicated that losses were significantly higher in locally made hives than in modern ones. This finding underscores the need to explore further how indigenous hive types and management practices influence the rate of losses due to pests, pathogens, and other factors.

The survey also revealed that migratory beekeepers experienced considerably lower losses overall than those who did not migrate. This pattern was consistent in Ethiopia, but not in Kenya and Benin, where losses were similar between migratory and non-migratory beekeepers. Across the globe, the impact of migration is also inconsistent. In the USA [91], Austria (in 2019) [92] and across Europe (in 2018 and 2020) [5,7], a lower loss rate was observed in migratory beekeeping than in non-migratory beekeeping, whereas an opposite trend was observed in South Africa [26] and Europe (in 2019) [5]. These varying results could be explained by differences in the quality and/or quantity of forage resources available to migratory and stationary colonies within their respective landscapes as well as migration conditions.

Regarding the significance of supplementary feeding and/or provision of water for colony wellbeing, our survey results did not show a clear effect. Over 70% of the interviewed beekeepers did not feed their colonies at the onset of swarming to facilitate colony establishment and/or to stimulate comb construction and population build-up, but did so after honey harvest when colonies enter the lean season. Since these periods are very different in terms of colony food requirements, it will be important in future to analyze separately the impact of supplementary feeding with emphasis on its quality in terms of carbohydrate and protein sources, for these two periods. For example, in Kenya and Benin, 8.4% and 7.1% of respondents, respectively, used remains of honey in beeswax to feed their colonies. In contrast, in Ethiopia and Rwanda, where feeding was most prevalent, various carbohydrate sources were used (e.g., sugar syrup, Bee Fonda, as well as juices from some local fruits) as well as pollen substitutes (e.g., flours of cassava, maize, sorghum, roasted pea, bean, soya, and/or barley). The quality protein and carbohydrate substitutes and their ratios are known to affect the reproduction and viability of the honey bees, as a result of their poor colony performance [93–98]. Similarly, the impact of water provision at the onset of swarming varied between the countries, but it did not affect the colony loss rate in Kenya, whereas beekeepers in Benin who provided water to their colonies had higher losses than those who did not. The latter finding in Benin could be a result of water contamination in a way that spreads harmful agents or chemicals between the colonies [99], but is surprising and requires further investigation.

Sociological factors, in particular education in beekeeping, also correlated with colony loss rate in countries such as Liberia, Nigeria, Rwanda, and Ethiopia. These countries recorded considerably lower losses (9.7–18.6%) and had more beekeepers who received training on best beekeeping practices (45–80.6%), compared to Cameroon, which recorded a 27.9% loss rate while only 26% of respondents in this country received training in beekeeping (Fig 1). These findings align with previous reports indicating a lack of training in bee farming in Cameroon [100]. Overall, these results underscore the need for improved beekeeping education to enhance the management of risk factors affecting colony health and productivity in this country.

## Conclusions

This pioneering study provides a first examination of managed honey bee colony loss rates and identifies several key risk factors within nine Sub-Saharan African countries during the active beekeeping season. Despite the sample size covering only one percent of all estimated beekeepers in each participating country, the implications of this research are significant, offering new insights into the challenges that the apicultural sector faces in this continent. The findings underscore critical risk factors that potentially threaten the sustainability of beekeeping, including pest and disease management, environmental stressors, and beekeeping practices. These challenges highlight the vulnerability of the apicultural sector in Africa, which is vital for pollination services that support agriculture and biodiversity as well as securing the livelihoods of beekeepers’ communities. Given the study's findings, there is a pressing need for ongoing, regular, long-term monitoring of honey bee colony losses in Africa and the variables influencing them. These initiatives across the continent will help in understanding broader patterns and causes of bee colony declines and facilitate the development of targeted interventions to mitigate these losses to ensure a sustainable apicultural sector in Africa. As the global importance of pollinators continues to gain recognition, enhancing the stability of honey bee colonies in Africa becomes both a continental priority and a global one.

## Supporting information

Table S1 The questions asked in the survey for 2022/2023; the asterisks indicate mandatory questions.
(DOC)

S1 file Raw data.
(RAR)
