# Supplementary material for: Honey bee colony losses and causes during the active beekeeping season 2022/2023 in nine Sub-Saharan African countries
Source: PLoS One. 2025 May 19;20(5):e0322489. doi: 10.1371/journal.pone.0322489 (PMC12088056; doi:10.1371/journal.pone.0322489)
Supplement: Table S1 — The questions asked in the survey for 2022/2023; the asterisks indicate mandatory questions. (DOC) [file pone.0322489.s001.DOC]

**Table S1**. **The questions asked in the survey for 2022/2023; the asterisks indicate mandatory questions.**

| **Q1.** Personal information from the beekeeper  Name: ____________________________________  Phone number: ____________________________________  Email: ____________________________________ | | | | |
| --- | --- | --- | --- | --- |
| **Q2*** Do you cultivate honey bees as:    o Professional (beekeeping is the only source of income)    o Semi-professional (beekeeping is not the only source of income)    o Hobbyist (beekeeping is only for personal or family consumption) | | | | |
| **Q3*** To describe the location of your apiary, please indicate:  a)  The name of the region/state/county where you keep your bees?_____  b)     The name of a city/town/village near your apiary? _______  c)     The GPS coordinates (if possible) _______ | | | | |
| **Q4***Have you received any training on best beekeeping practices? | | | No | Yes |
| **Q5*** Do you catch bee swarms for operation expansion? | | | No | Yes |
| **Q6*** Do you practice queen rearing for operation expansion? | | | No | Yes |
| **Q7*** How many production colonies did you have before the swarming season on 1^st^ September 2022? _______ | | | | |
| **Q8*** How many production colonies did you catch or purchase during the swarming season between September 2022-June 2023? _______ | | | | |
| **Q9*** How many production colonies did you sell or give away during September 2022-June 2023? _______ | | | | |
| **Q10*** How many production colonies did you have after honey harvest on 30^th^ June 2023? _______ | | | | |
| **Q11*** How many production colonies did you lose during September 2022-June 2023? _______ | | | | |
| **Q12*** Out of total number of colonies lost, how many were lost due to:  a)  Issues beyond the beekeeper’s control (e.g. theft, bush fire, strong wind, falling trees, drought, cold, etc.) _______  b)  Absconding _______  c)  Starvation _______  d)  Pesticide poisoning _______  e)  Queen problems (queen loss or drone-laying queens) _______  f)  Pests/pathogens _______  g)  Other factors (enter explanation): _______  h)   Unknown symptoms _______ | | | | |
| **Q13*** If beekeepers consider losses due to pests/pathogens identified in Q12, they were asked to choose among the known pests and pathogens reported in Africa  O American foulbrood disease o European foulbrood disease o Chalkbrood disease o Sacbrood disease o Nosema disease o Viruses o Wax moth o *Varroa destructor* mite o Large hive beetle o Braula fly o Small hive beetle o Honey badger o Birds (bee-easters) o Ants o Wasps | | | | |
| **Q14*** Did you treat your colonies against any pest/disease/parasite identified in **Q13**? | No | Yes | If yes, please indicate the mode of treatment _______ | |
| **Q15*** Did you migrate any colonies from September 2022 to June 2023? | No | Yes | If yes, for what purpose:  O Honey  O Crop pollination  O or both | |
| **Q16*** Did you practice supplemental feeding before swarming and/or after the honey harvesting period during September 2022-June 2023? | No | Yes |  | |
| **Q17*** Did you keep your colonies in an area where there is water scarcity during September 2022-June 2023? | No | Yes | If yes, did you provide water to the colonies before swarming and/or after the honey harvesting period during September 2022-June 2023?  Yes______ No ______ | |
| **Q18***How many colonies were lost in Q11 from:  O Locally made hives without any frames______  O Modern hives with movable top bars/frames ______ | | | | |
| **Q19** To calculate increases and decreases in the number of swarm catches over the past three years: How many swarms did you catch?   o This year 2022/2023? If no idea, write “I don’t know”. ______   o Last year 2021/2022?  If no idea, write “I don’t know”.  ______  o Two years ago 2020/2021?  If no idea, write “I don’t know” ______ | | | | |
